# Codeine and Tramadol Use in Athletes: A Potential for Abuse

**DOI:** 10.3389/fphar.2021.661781

**Published:** 2021-06-10

**Authors:** Thomas Zandonai, Mónica Escorial, Ana M. Peiró

**Affiliations:** ^1^ Sports Research Centre, Department of Sport Sciences, Miguel Hernández University of Elche, Elche, Spain; ^2^ Department of Pharmacology, Paediatrics and Organic Chemistry, Miguel Hernández University of Elche, Elche, Spain; ^3^ Neuropharmacology on Pain and Functional Diversity (NED), Institute of Health and Biomedical Research of Alicante (ISABIAL Foundation), Alicante, Spain; ^4^ Pain Unit, Department of Health of Alicante-General Hospital, Alicante, Spain

**Keywords:** pain, athletes, doping, health, sports

## Exercise and Pain in the Sports Population

Analgesic use by athletes is common (Overbye, 2020). It has been observed that athletes used analgesics up to four times more often than their age-matched general population (Holgado et al., 2018a). Notably, codeine and tramadol are not included in the WADA list of prohibited substances in sport (WADA, 2021a). Their use could be an attempt to compensate for fatigue, pain, and inflammation caused by injuries (Hainline et al., 2017) or overtraining (Grandou et al., 2020). It has been described that this abuse can be related to false expectations of performance improvement (Lundberg and Howatson, 2018). A 2017 study reported a small but a significant performance-enhancing benefit of tramadol in 20-min cycling time trial (Holgado et al., 2018b), however, with nonsignificant results being reported in subsequent studies (Experiment 2 of Holgado et al., 2018b; Bejder et al., 2019; Zandonai et al., 2021). To the best of our knowledge, no studies have investigated codeine’s effects on exercise performance. Tramadol used by athletes may be linked to its analgesic and mood-enhancing effects (Grond and Sablotzki, 2004; Chaparro et al., 2013; Bastami et al., 2014); the reinforcement of euphoric symptoms may contribute to the onset of addiction. The suppression of pain sensation and increased pain tolerance and improved mood may be sufficient to motivate an athlete to push harder leading to small performance gains. However, tramadol and codeine may have side effects and health risks such as nausea, dizziness, drowsiness, and difficulty in concentrating (Poulsen et al., 1996; Vazzana et al., 2015). Scientific evidence regarding the impact of use and abuse on the sports population’s health is scarce (Zideman et al., 2018), much less, with a sex- or gender perspective. We present an overview evidencing the athletes’ use of codeine and tramadol and their potential abuse and addiction.

## Athletes’ Codeine Use

Codeine or 3-methylmorphine is one of the most commonly used opiates for managing moderate pain and relieving mild coughs (Seif-Barghi et al., 2015; Kaye et al., 2019). It is used by athletes and was included in the 2021 World Anti-Doping Agency monitoring list (WADA 2017; 2021b). Codeine is mainly metabolized into codeine-6-glucuronide by uridine diphosphate glucuronosyltransferase enzymes (*UGT2B7*). Thus, approximately 10% of an oral dose of codeine is converted into morphine by the cytochrome *P450 2D6* enzyme (*CYP2D6*), which can lead to a positive urine test (Seif-Barghi et al., 2015). This enzyme is also responsible for the metabolism of many other opioids and the management of concomitant pain drugs such as anticonvulsants, anxiolytics, or antidepressant drugs (Kaye et al., 2019). Following codeine administration, the initial morphine levels in the brain, and consequently initial central effects, may be determined by the individual’s brain *CYP2D-*mediated metabolism (Konno and Sekiguchi, 2018). In this way, genetic variation in the *CYP2D6* gene directly affects enzyme activity and contributes to the inter-individual variability in codeine effects observed across ethnicities (Poulsen et al., 1996). For example, it has been estimated that 3% of the Caucasian population is associated with ultrarapid metabolism, making those individuals more susceptible to adverse effects from some prodrugs such as codeine and tramadol (Drewes et al., 2013). In this sense, the risk of codeine abuse becoming a gateway into drug addiction can be elevated depending on receptors’ distribution in the mesolimbic system, which serves to modulate dopaminergic activity and, thus, reinforces these reward circuits (Listos et al., 2019). However, there are no randomized controlled trial studies analyzing codeine’s effects on exercise.

As a matter of fact, *CYP2D6* is an actionable marker for codeine tolerability according to the Clinical Pharmacogenetics Implementation Consortium guideline (Palareti et al., 2016). Besides, regarding gender, a higher *CYP2D6* expression and activity among extensive metabolizers have been observed in women (Zhang et al., 2011), suggesting a biological difference (Hagstrom et al., 2021). In fact, an increase in *CYP2D6* activity has been observed in pregnant women. This has been related to a change in the expression of the heterodimer pattern in the liver tissue. Further studies are needed to understand the underlying biological differences.

## Athletes’ Tramadol Use

Another analgesic commonly used by athletes is tramadol (Holgado et al., 2018b; Baltazar-Martins et al., 2019). Baltazar-Martins et al. estimated a prevalence of tramadol use in sports competition of 1.4% based on the analysis of 9.851 urine samples (Baltazar-Martins et al., 2019). The maximum consumption was found in cycling (65%), followed by triathlons (8%), and rowing (6%). Those data are in agreement with those from other WADA-accredited laboratories, thus confirming a prevalence of tramadol use between 49.5% (WADA, 2018) and 61% (Baltazar-Martins et al., 2020). There is a wealth of literature on the effectiveness of tramadol in the therapy of musculoskeletal pain (Chaparro et al., 2013). Its effects on exercise performance are still being debated despite tramadol having been banned in cycling competition in 2019 (Zandonai et al., 2021). However, there are only four randomized controlled trial studies that have investigated the effects of tramadol on exercise. Holgado et al. (2018b) studied whether a single oral dose of 100 mg (vs. placebo) modifies performance during a 20-min cycling time trial and whether sustained attention would be impaired during cycling after tramadol intake. Results of this study revealed that tramadol improved 20-min cycling time trial performance by ∼5%, but when participants performed a sustained attention task during exercise, performance improvement could not be confirmed (Holgado et al., 2018b). A recent study by Bejder et al. (2019) tested the effects of 100 mg of tramadol on a constant work rate (1 h at 60% of peak power), followed by a 15-km time trial, using 16 male participants. These authors concluded that under these conditions, tramadol neither improved physical performance nor impaired cognitive performance. Finally, Zandonai et al. (2021) tested 29 male participants in a preloaded cycling time trial after they had ingested 100 mg of tramadol [vs. placebo and paracetamol (1.5 g)]. Participants performed the psychomotor vigilance task at rest and a sustained attention to response task during the 60 min of exercise. The results showed higher mean power output during the 20-min time trial in the tramadol athletes in comparison to paracetamol athletes, but no significant difference was reported between tramadol and placebo (or paracetamol vs. placebo). The authors found an increased behavioral and neural efficiency at rest for tramadol, but not the proposed ergogenic or cognitive (harmful) effect of tramadol (vs. placebo) during self-paced high-intensity cycling exercises. Interesting to note is that the four randomized controlled trial studies previously mentioned recruited and investigated females as only 9.8% of their participants. Research studies on tramadol (vs. placebo) administration on females are needed. A summary of these studies’ results is shown in Table 1.

**TABLE 1:** Study design and intervention characteristics of the included studies which investigated the effects of tramadol.

Study (year)/	Study design	Participants	Dose administered (mg)	Exercise and cognitive testing	Outcomes
Holgado et al. (2018b) (Experiment 1)	Randomized cross-over double-blind placebo-controlled.	Healthy volunteers *n* = 19 males and 9 females; age 26 (6) years; VO2max: 49 (7) ml/min/kg [mean (SD)].	Tramadol (100 mg) vs. placebo capsules/120 min before time trial	20-min of time trial on a cycle ergometer.	Average time trial power output was higher in the tramadol vs. placebo condition (tramadol: 220 watt (W) vs. placebo: 209 W; *p* < 0.01).
Holgado et al. (2018b) (Experiment 2)	Randomized cross-over double-blind placebo-controlled.	Healthy volunteers *n* = 28 males; age 25 (5) years; VO2max: 54 (6) ml/min/kg [mean (SD)].	Tramadol (100 mg) vs. placebo capsules/120 min before time trial	20-min of time trial on a cycle ergometer; a visual oddball task during time trial.	No significant difference between tramadol and placebo was observed in the average power output (tramadol: 234 W vs. placebo: 230 W). No behavioral differences were found between conditions in the oddball task.
Bejder et al. (2019)	Randomized cross-over double-blind placebo-controlled.	Healthy volunteers *n* = 16 males; age 26 (5) years; VO2max: 64 (6) ml/min/kg [mean (SD)].	Tramadol (100 mg) vs. placebo capsules/160 min before time trial	Preloaded 60 min of constant work rate at 60% of peak power and 16-km of time trial on a cycle ergometer; visuo-motor tracking and math tasks were completed during the 60-min preload by 10 participants.	No significant difference between tramadol and placebo was observed in the average power output (tramadol: 298 W vs. placebo: 294 W) and performance time (tramadol: 1,474 s vs. placebo: 1,483 s). Tramadol did not impair the ability to complete certain cognitive and fine motor task performance during submaximal exercise.
Zandonai et al. (2021)	Randomized cross-over double-blind placebo-controlled.	Healthy volunteers *n* = 29 males; age 26 (7) years; VO2max: 53 (6) ml/min/kg [mean (SD)].	Tramadol (100 mg) vs. placebo capsules/120 min before time trial (1.5 g of paracetamol).	Preloaded of 40-min of constant work rate at 60% of the VO2max and 20-min of time trial on cycle ergometer. Sustained attention to response task (SART) during the all 60-min of exercise.	No significant difference between tramadol (227 W) and placebo (221 W) (only significant difference was found between tramadol and paracetamol (213 W) but no between paracetamol and placebo). No difference was shown in sustained attention to response task (SART) during the 20-min time trial.

Tramadol is a prodrug whose active metabolite, *O-*desmethyltramadol, has analgesic properties (Palareti et al., 2016). It has a dual mechanism of action: as an m-opioid receptor agonist and as a serotonin and norepinephrine reuptake inhibitor (Grond and Sablotzki, 2004). Genetic variations could condition different pharmacokinetic levels due to modifications on drug absorption, distribution, metabolism, or excretion. In the area of opioid abuse, the most studied gene variant is *A118G* linked to the *OPRM1* gene, which is the main receptor for endogenous opioids such as beta-endorphin for analgesia, mood enhancers, and addiction effects (Peiró, 2018). When it is activated, reinforcement of euphoric symptoms appears and could contribute to the onset of addiction. This variant is characterized by the replacement of adenine with guanine at position *118 (rs1799971).* It has been observed that the presence of heterozygotes (A/G) affects its affinity for beta-endorphin, increasing it (Oslin et al., 2003) and, thus creating need for modified dosage of opioids. In the case of tramadol, it is known that the genes of the cytochrome *P450* enzymes (*CYP2D6 and CYP3A4*) influence their metabolism and potential for hepatotoxicity. The analgesic potential of tramadol can be influenced by the genetics of an individual’s CYP genes due to its need for hepatic bioactivation by *CYP2D6* (Palareti et al., 2016). To this point, slow metabolizers experience little conversion to the active M1 opioid metabolite and people with a high metabolic profile, or ultrametabolizers, have the greatest opioid analgesic effects. This implies a complex nature in the genotype × phenotype interaction.

Recent research has shown that tramadol has a more pronounced potential for abuse, and it has therefore been reclassified as a controlled substance in several countries (Birke et al., 2018). What is more, *OPRM* has been widely studied for its association with a variety of drug addiction and pain sensitivity phenotypes (Mague et al., 2009), including sex-specific effects that, moreover, could modulate the *COMT* genotype’s effect on synaptic dopaminergic concentrations and emotion modulation (Hill et al., 2018). Thus, genetic variations together with environmental factors are implicated in the development of opioid misuse and addiction (Taqi et al., 2019). The fact that age, sex/gender, comorbid mental disorders, type of administration, type of opioids, and body weight significantly affect rates of misuse suggests that men and women may differ in their responses to opioids for pain relief. However, the role of these factors is not usually evaluated in the prescription of opioids, such as tramadol for pain control (Pisanu et al., 2019). Knowledge of genetic variants could help optimize treatment with tramadol to achieve better pain control (Peiró, 2018) and avoid its adverse effects, such as abuse (Bastami et al., 2014; Holgado et al., 2018b; Lundberg and Howatson, 2018).

## Sex-Perspective Exercise and Analgesics Pattern

Sex-/gender-related differences in pain perception and analgesic response have been studied and show mixed results (Sorge and Strath, 2018). Sports research was previously conducted without explicit reference to the sex of the participants, the majority of whom were male (Costello et al., 2014). Also, a recent review about hormone cycle influence on sports performance revealed only poor quality studies present in the literature (McNulty et al., 2020). Physical exercise seems to reduce of menstrual pain intensity (Armour et al., 2019), and oral contraceptive pill use experiences high prevalence among athletes (Martin et al., 2018). What is more, there is much evidence to suggest that gender is an important factor in the modulation of pain. Gender differences in pharmacological therapy and nonpharmacological interventions have also been reported (Pieretti et al., 2016), though not applied to the sport area. Therefore, this lack in sports-related data about the effects of drug use between women and men athletes should be considered in order to reduce gender differences and to individualize preventive strategies. Moreover, the marked female predominance among some pain subtypes is often attributed to a psychosocial predisposition.

In this way, sex may modify drug outcomes through pharmacokinetics and pharmacodynamics. For example, it has been described that men have a greater gastric motility and intestinal transit than women (Kimura and Higaki, 2002). This is important because the gastrointestinal transit rate can affect the plasma concentration and absorption of oral drugs. Men also have been shown to have a higher hepatic expression of p-glycoprotein, an ATPase transporter protein also known as a multidrug resistance protein, leading to faster transport and a shorter elimination half-life of drugs (Ofotokun, 2005). Females normally have a higher percentage of body fat, which can affect the volume of distribution of certain drugs, especially opioids and benzodiazepines. In addition, a slower clearance of drugs has been reported in women than men. Here, pregnancy is also a factor, as it can alter the pharmacokinetics of drugs due to changes in the pharmacokinetics processes (Soldin and Mattison, 2009). Science needs to focus on sex differences in neurobiological processes, specifically those related to hormone immune regulation or brain networks that could directly influence clinical pharmacology.

## Conclusion and Further Directions

The inconclusive nature of findings on the effects of analgesics such as tramadol and codeine on exercise is evident. Data need to be collected on the prevalence in the use of codeine and tramadol in both male and female athletes, their effects on exercise performance, and the impact of health. Here, pharmacogenetics could offer a means to identify potential biomarkers that can predict individual analgesic response and tolerability (Peiró, 2018). Knowing more information about the drug–exercise influences may lead to improved athlete safety, with potential implications for clinical outcomes, including addiction. Indeed, the pharmacokinetics studies are usually done in non-stressful conditions at rest, as opposed to during exercise, and with healthy and nonmedical volunteers.

In conclusion, there is an urgent need to conduct clinical trials on the use of opioid medications for pain, such as codeine and tramadol, in men and women under exercise conditions. Understanding how certain, possibly gender-based risk factors contribute to pain experience in the sport area may be an important step toward lowering the prevalence of analgesic abuse.
